# Systematic review on the management of term prelabour rupture of membranes

**DOI:** 10.1186/s12884-023-05878-x

**Published:** 2023-09-08

**Authors:** Lucia Ramirez-Montesinos, Soo Downe, Annette Ramsden

**Affiliations:** 1 Imperial College NHS Trust, St. Mary’s Hospital, Praed Street, W2 1NY London, UK; 2 Department of Midwifery, University of Central Lancashire, Brook Building, PR1 2HE Preston, UK; 3 Library services, University of Central Lancashire, PR1 2HE Preston, UK

**Keywords:** Prelabour rupture of membranes, PROM, Induction, Expectant management, Vaginal examination, Normal birth, Chorioamnionitis

## Abstract

**Introduction:**

Prelabour rupture of membranes at term affects approximately 10% of women during pregnancy, and it is often associated with a higher risk of infection than when the membranes are intact. In an attempt to control the risk of infection, two main approaches have been used most widely in clinical practice: induction of labour (IOL) soon after the rupture of membranes, also called active management (AM), and watchful waiting for the spontaneous onset of labour, also called expectant management (EM). In addition, previous studies have demonstrated that vaginal examinations increase the risk of chorioamnionitis. However, the effect of vaginal examinations in the context of prelabour rupture of membranes have not been researched to the same extent.

**Methods:**

This systematic review analyses and critiques the latest research on the management of term prelabour rupture of membranes, including the effect of vaginal examinations during labour, with a focus on the outcomes of both normal birth, and chorioamnionitis. Due to its complexity, three research questions were identified using the PICO diagram, and subsequently, the results from these searches were combined. The systematic review aimed to identify randomised controlled trials (RCTs) and observational studies that compared active vs expectant management, included number of vaginal examinations and had chorioamnionitis and/or normal birth as outcomes. The following databases were used: MEDLINE, EMBASE, Maternity and Infant care, LILACS, CINAHL and the Cochrane Central Register of Controlled trials. Quality was assessed using a tool developed especifically for this study that included questions from CASP and the Cochrane risk of bias tool. Due to the high degree of heterogeneity meta-analysis was not deemed appropriate. Therefore, simple narrative analysis was carried out.

**Results:**

Thirty-two studies met the inclusion criteria, of which 27 were RCTs and 5 observational studies. The overall quality of the studies wasn’t high, 15 out of the 32 studies were deemed to be low quality and only 17 out of 32 studies were deemed to be of intermediate quality. The systematic review revealed that the management of term prelabour rupture of membranes continues to be controversial. Previous research has compared active management (Induction of labour shortly after the rupture of membrane) against expectant management (watchful waiting for the spontaneous onset of labour). Although previous studies have demonstrated that vaginal examinations increase the risk of chorioamnionitis, no prospective studies have included an intervention to reduce the number of vaginal examinations.

**Conclusion:**

A RCT assessing the consequences of active management and expectant management as well as the effect of vaginal examinations during labour for term prelabour rupture of membranes is necessary.

**Supplementary Information:**

The online version contains supplementary material available at 10.1186/s12884-023-05878-x.

## Introduction

Spontaneous rupture of membranes (SROM) is a normal physiological event. In about 10% of the population, it happens before labour starts [1]. It is believed that prelabour rupture of membranes increases the risk of infection and therefore induction of labour is recommended in an attempt to reduce such risk [2]. However, there is controversy about whether the induction reduces that risk. Moreover the risk of infection is always present, even when the membranes are intact. One of the downfalls of the routine induction of labour is that it limits the potential for women and her infant to experience a normal/physiological birth and its long term benefits.

Therefore, the management of prelabour rupture of membranes has been an issue of debate since the 1960’s and the pendulum has swung between inducing labour as soon as possible in an attempt to reduce the risk of infection, and giving women time to start labour spontaneously in an attempt to increase the chances of having a physiological birth and reduce the risk of caesarean section that can be associated with the induction of labour for women with prelabour rupture of membranes [3]. A Cochrane systematic review published by Middleton et al. [4] found an increase in maternal infectious morbidity (chorioamnionitis and/or endometritis combined) for women who had expectant management following term prelabour rupture of membranes, and their infants were more likely to have definite or probable early-onset neonatal sepsis combined. However, importantly for this review, Middleton et al. [4] found no statistically significant differences for caesarean section, serious maternal morbidity and mortality, definite neonatal sepsis alone, or perinatal mortality.

In addition, vaginal examinations have been known to be associated with an increased risk of chorioamninionitis. One of the first studies to highlight this issue was carried out by Schutte et al. [5]. They discovered that what was more significant was the length of time between the first vaginal examination and the birth rather than the time between the rupture of membranes and the birth. Further studies have demonstrated that vaginal examinations increase the risk of chorioamnionitis [6]. However, since vaginal examinations are a very common procedure in clinical practice, these are often overlooked and its effects have not been extensively researched in the context of prelabour rupture of membranes.

The incidence of induction of labour keeps rising, increasing in England from 22% in 2011-2012 to 33% in 2021-2022 [7]. Prelabour rupture of membranes at term is a common cause for routine induction of labour due to national guidelines’ recommendations [2]. The aim of this systematic review is to identify, evaluate and synthesise the results from observational and RCTs studies over the past three decades that compare active vs expectant management, that include vaginal examinations, and that had chorioamnionitis and/or normal birth as outcomes.

## Methods

This section of the paper outlines the process that was followed to identify the primary research studies that answered the research questions. Due to its complexity, three research questions were identified using the PICO diagram and subsequently, the results from these searches were combined. The research questions are:

1) For term prelabour rupture of membranes, is expectant management associated with a lower rate of chorioamnionitis compared to active management? 2) For term prelabour rupture of membranes, is expectant management associated with a higher rate of normal birth compared to active management? 3) For term prelabour rupture of membranes, are vaginal examinations associated with chorioamnionitis?

The systematic review aimed to identify randomised controlled trials (RCTs) and observational studies that compared active vs expectant management, vaginal examinations and had chorioamnionitis and/or normal birth as outcomes. The following databases were used: MEDLINE, EMBASE, Maternity and Infant care, LILACS, CINAHL and the Cochrane Central Register of Controlled trials (CENTRAL). The systematic review was last updated in November 2019. No time limit was set on the searches as the aim was to identify all research studies that met the inclusion criteria to see how the management of prelabour rupture of membranes had evolved over time. The three research questions stated above refer to term pregnancy (36 weeks or more). The searches were performed initially without specifying gestational age but were subsequently screened manually for term pregnancy. This is because it was the approach that identified more studies. The studies that referred to less than 36 weeks gestation were excluded manually. It was decided to include papers in all different languages. Therefore, all the published studies that met these criteria were listed regardless of language. However, only papers written in English, Spanish or French were read and analysed. The inclusion criteria are outlined on Table 1.

**Table 1:** Inclusion and exclusion criteria

Number	Inclusion criteria	Exclusion criteria
1	Quantitative primary research (RCT or observational)	Non-primary research
2	Prelabour rupture of membranes (PROM)	Studies not focused on PROM
3	Gestational age $\ge$36 weeks	Gestational age <36 weeks
4	Studies that compare active vs expectant management	Other comparisons
5	Studies that analyse the effect of vaginal examinations in the context of PROM	Studies that do not analyse the effect of vaginal examinations in the context of PROM
6	Papers published in all languages	Not applicable
7	Papers published since the start of the database (No time limit)	Not applicable

### Quality assessment

There are several published tools to aid the quality assessment of research studies, as well as different tools depending on the type or methodology of the research. The quality assessment process carried out for this review is based on a synthesis of both the CASP tools [8–10] and the Cochrane risk of bias assessment tool developed by Higgins et al. [11]. This was because this systematic review included observational studies, as well as randomised controlled trials. Therefore, 13 questions were used for the RCTs and 12 questions for the observational studies. Tables 2 and 3 present the questions that were used to assess the quality of the RCT and observational studies respectively, and which published tool they were conceptually drawn from.

Although the risk of bias assessment tool developed by Higgins et al. [11] is well known and well accepted by the academic community to assess the quality of RCTs, It was decided to add some of the questions and concepts developed by the Critical Appraisal Skills Programme to complement it because they would contribute to assess the quality of all the studies included in this systematic review. The implications of this choice are that two similar lists of questions were created that made the process of assessing the quality of the studies less complicated, without compromising the quality assessment. Both, the Cochrane risk of bias assessment tool and the Critical Appraisal Skills Programme are deemed good tools to assess the quality of the studies.

**Table 2:** Quality assessment questions for RCT studies

Question Number	Question	Original tool
1	Did the study address a clearly focused issue?	CASP
2	Did the study clearly stated primary and secondary outcomes?	Cochrane
3	Did the study have enough statistical power?	Cochrane
4	If it was a trial, was the assignment of patients to treatments randomised?	CASP
5	Were participants or staff blinded?	CASP
6	Was there any blinding for the outcome assessment?	Cochrane
7	Were the characteristics of the groups similar?	CASP
8	Were the groups treated differently (except for the intervention)?	CASP
9	Were all the participants accounted for at its conclusion?	CASP
10	Number of participants with missing outcome data	Cochrane
11	Selective reporting?	Cochrane
12	Other important bias identified?	Cochrane
13	Were all the clinically important outcomes considered?	CASP

Furthermore, the results from the systematic review are in agreement with a recent Cochrane systematic review published by Middleton et al. [4] in that the quality of most studies in this topic is generally low. Since there was a high degree of heterogeneity in the outcomes to be measured, it was not possible to do meta-analysis. All the studies that were found in the searches that met the criteria are presented in Table 4, and no studies were omitted due to their quality. Therefore, there is no bias in reporting.

A total of 13 questions were to be answered by the RCTS and 12 questions for the observational studies. All questions were deemed equaly important and had the same weight. Therefore, all studies were given a score between 0 and 12 or 13, in which 0 indicated very poor quality and 12-13 extremely good quality and then a percentage was obtained, for example 8/13 (61.5%). It was decided a priori that studies that scored less than 40% were considered low quality, studies that scored between 41% and 79% were considered intermediate quality and studies that scored 80% or more were considered of high quality. In order to maintain consistency and rigor, in the case of RCT studies where it was not clear what the primary and secondary outcomes were or in cases where these were not stated, these studies were given a score of 0 as an answer to question: “Were the primary and secondary outcomes clearly stated?”. Also in the case of RCT studies, if the randomisation system used was either not stated or the allocation to treatment was done by the day of the week, or the number of the hospital number or by means of sealed envelopes, these studies were given a score of 0 as an answer to the question “In the case of RCT, was the allocation to treatment randomised?”.

**Table 3:** Quality assessment questions for observational studies

Question Number	Question	Original tool
1	Did the study address a clearly focused issue?	CASP
2	Did the study clearly stated primary and secondary outcomes?	Cochrane
3	Did the study have enough statistical power?	Cochrane
4	Were participants or staff blinded?	CASP
5	Was there any blinding for the outcome assessment?	Cochrane
6	Were the characteristics of the groups similar?	CASP
7	Were the groups treated differently (except for the intervention)?	CASP
8	Were all the participants accounted for at its conclusion?	CASP
9	Number of participants with missing outcome data	Cochrane
10	Selective reporting?	Cochrane
11	Other important bias identified?	Cochrane
12	Were all the clinically important outcomes considered?	CASP

## Results

**Fig. 1:**
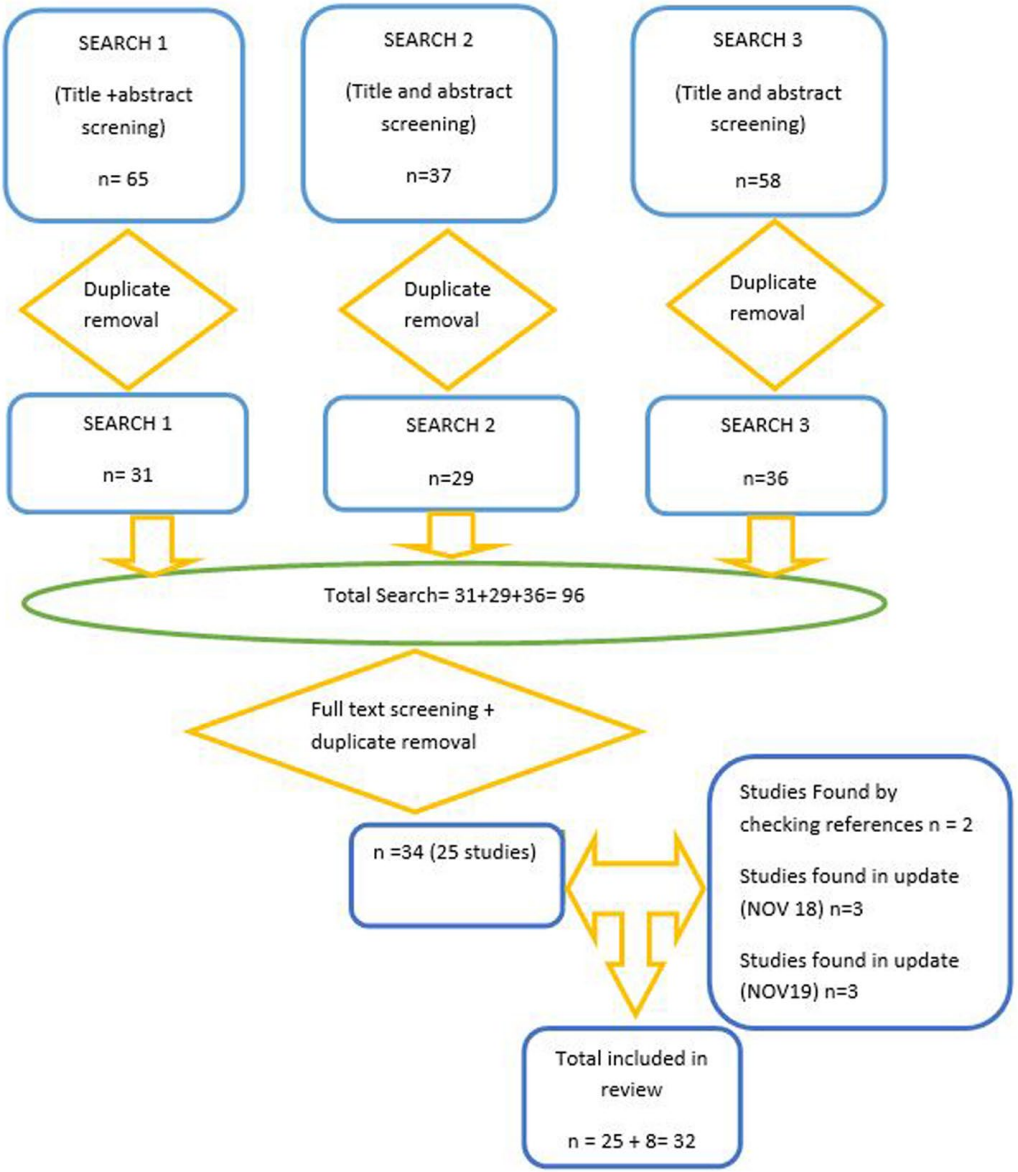
Summary of results from the three searches

In total, there are 32 studies included in this review after the final search in November 2019, 27 studies were RCT (Randomised controlled trials) or quasi-randomised and 5 were observational studies [12–16]. In this systematic review, what is understood by randomised controlled trial is a study that has a truly random method of allocating participants to the different treatment groups, such as a random list of computer generated numbers or a computer that does the randomisation online, which means it cannot be predicted which treatment group the participant will be allocated to. On the other hand, a quasi-randomised trial, is one in which the allocation of participants can be easily predicted, because the study uses a method of allocation that is not random, for example, when the allocation of participants is based on the last digit of the date of birth, or the last digit of the medical record number, or odd numbers are allocated to group 1 and even numbers to group 2. Using these easily predictable methods to allocate participants to different treatment groups can introduce selection bias into the study. In this systematic review several studies were deemed to be quasi-randomised controlled trials, such as [17–22]. Figure 1 summarises the results obtained through the three searches mentioned earlier. This figure shows the number of papers that were relevant and met the inclusion criteria prior to assessing their quality.

The 32 studies were undertaken all over the world, the TERMPROM was an international multicentre study that was carried out in 6 countries (Canada, UK, Australia, Sweden, Denmark, and Israel), six were undertaken in Europe, five in the USA, one in Canada, one in South America, two in Africa, six in Middle East, and four in Asia.

With regard to the length of time of the expectant management, out of these 27 RCT studies, two had expectant labour up to 12 hours, two studies compared IOL (induction of labour) at 12 hours vs IOL at 24 hours, 12 studies had EM (expectant management) up to 33 hours, three studies had an expectant arm up to 48 hours, three RCT had an expectant management up to 96 hours and five RCT did not state a time limit on the expectant management [21–25].The Supplementary file presents the RCT and observational studies found, organised in different tables according to the length of SROM (spontaneous rupture of membranes) in the expectant management.

The other aspect of study in these research studies was which agent/drug is associated with better clinical outcomes, across the included studies, the three drugs used to initiate labour were: Intravenous oxytocin, prostaglandings (PGE$_{2}$) and misoprostol (PGE$_{1}$). This systematic review, was not focused on the drugs that were used during the induction of labour but on the comparison between expectant and active management.

With regard to the primary outcome, the majority of studies focused on caesarean section or neonatal infection. No studies used “normal birth” or an equivalent term, or chorioamnionitis, as a primary outcome. Making the choice of primary outcomes included in this systematic review is one of the elements of originality. The majority of the studies were of poor quality and only four studies were scored 60% or more [3, 26–28]. The main problems were that the primary and secondary outcomes were not stated, the lack of definition of the outcomes, studies that are not properly randomised (i.e studies where the allocation could be predicted, for example allocation by the day of the week, or the number at the end of the case notes) or cases of selective reporting amongst other issues.

The small sample size, was another issue. With the exception of the TERMPROM trial, other studies whose primary outcome was neonatal infection were underpowered.

**Fig. 2:**
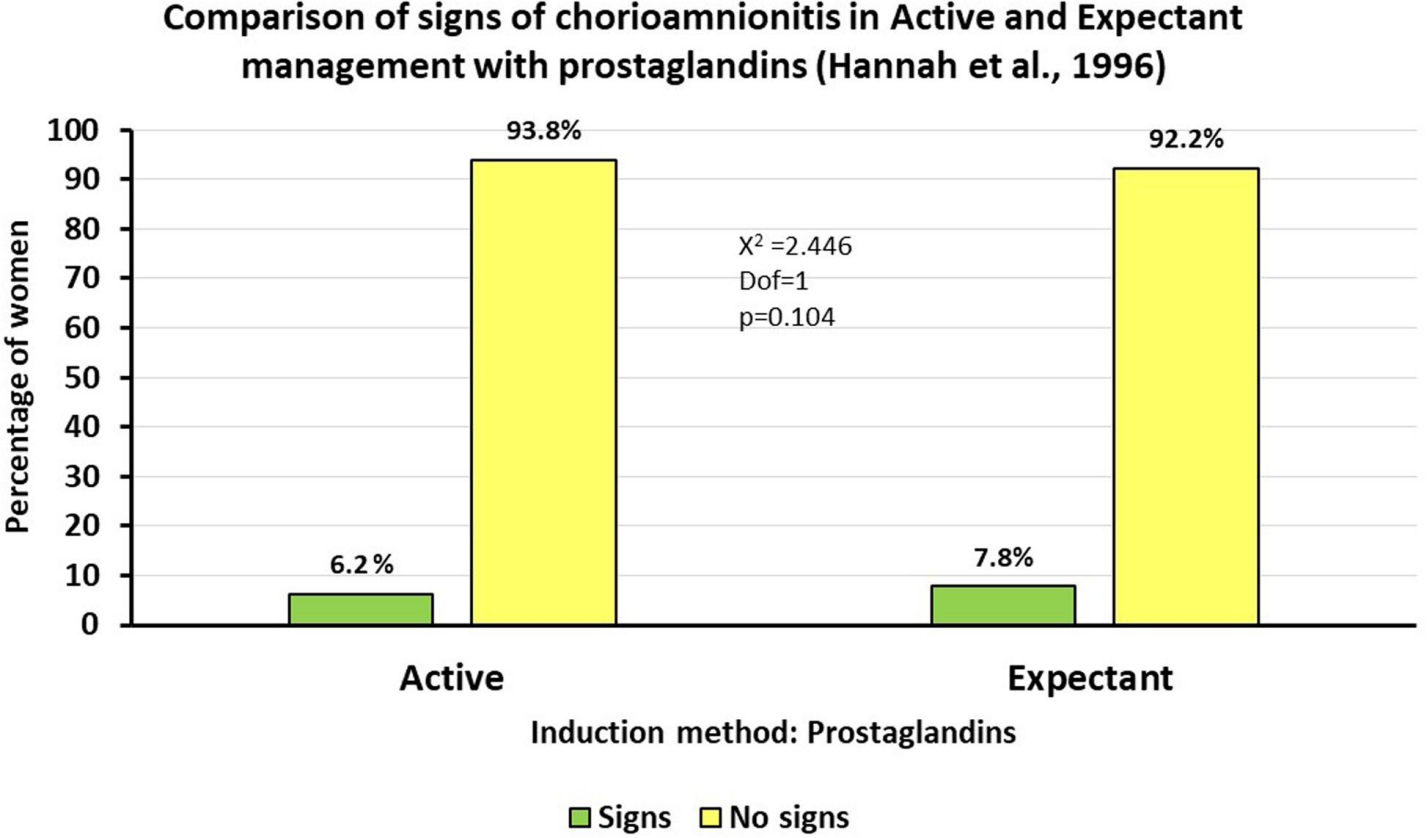
Chi-square test results on chorioamnionitis in TERMPROM study

In the TERMPROM study [27], the rate of chorioamnionitis was higher when women had expectant management in comparison to those who had active management and were induced with IV oxytocin. However, the study authors do not report that the difference in chorioamnionitis between Active management and Expectant management [78/1259 (6.2%) vs 99/1261 (7.8%)] when inducing with prostaglandins was not statistically significant ($X^{2}$=2.446, Dof=1; *p*=0.104). Figure 2 provides a graphical representation of these results.

The results found when inducing with intravenous oxytocin have been generalised, when, if labour is induced with prostaglandins, the difference is not significant.

This finding is important since nowadays labours are more likely to be induced with prostaglandins than with oxytocin alone. Therefore, the results that are relevant to current practice suggest that there may be less risk of chorioamnionitis associated with expectant management than has previously been assumed based on the TERMPROM results [27].

In terms of the mode of birth, as stated earlier no studies used “normal birth” or an equivalent term as primary outcome. However, the studies conducted by Grant et al. [26], and Natale et al. [28] had caesarean section as primary outcome, but only the study carried out by Grant et al. [26] had enough statistical power to address caesarean section as a primary outcome.

Grant et al. [26] compared active management (Inmediate induction of labour with IV oxytocin) with expectant management (up to 33 hours) and concluded that women allocated to the expectant management had fewer caesarean sections [38/219 (17.4% vs 25/225 (11.1%)] OR0.60; 95%CI 0.35 to 1.02; P=0.06] but the difference was not statistically significant.

**Fig. 3:**
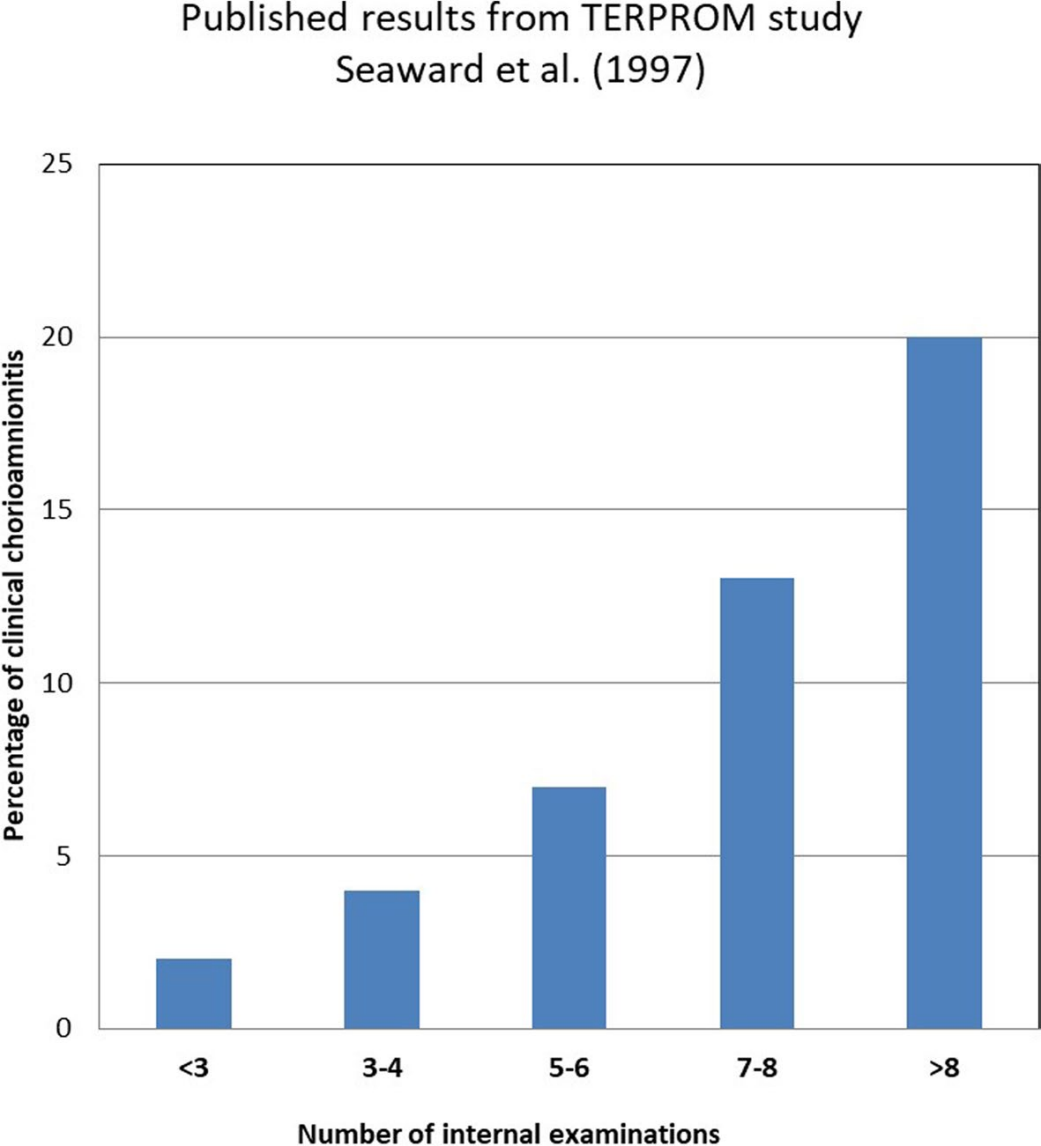
Relationship between number of VEs and chorioamnionitis

The number of vaginal examinations that women received during labour was not the primary focus in any of the included studies, although the TERMPROM study [6] highlighted that the number of vaginal examinations was the strongest correlator of chorioamnionitis. Figure 3 shows a graphical representation of the relationship between VEs and chorioamnionitis based on the data published by Seaward et al. [6].

The number of vaginal examinations during labour appeared to be retrospectively analysed in some studies [26, 27, 29]. While a secondary analysis performed by Seaward et al. [6] on the TERMPROM trial concluded that vaginal examinations were associated with higher rates of infection, none of the included studies prospectively included an intervention to try to minimise chorioamnionitis by reducing the number of vaginal examinations. The studies performed by Akyol et al. [29] and Grant et al. [26] reported the number of vaginal examinations, the former as a categorical variable and the latter as a continuous variable. Neither of them conducted any analysis to see if the number of vaginal examinations was associated with chorioamnionitis. Akyol et al. [29], Hannah et al. [27], and Grant et al. [26] analysed the number of vaginal examinations whereas the studies conducted by Natale et al. [28] and Ottervanger et al. [3] did not.

**Table 4:** Summary of studies included in the review

First author, year Country	Type of study	Total N	Intervention & comparison	Quality	Overall Quality
Poornima, 2011 India	RCT	N=100		5/13 (38.5%)	Low
Ray, 1992 USA	RCT	N=140	G$_{1}$: Immediate IOL with PGE$_{2}$	5/13 (38.5%)	Low
			G$_{2}$: Immediate IOL with oxytocin		
			G$_{3}$: Expectant up to 12h		
			G$_{1}$ & G$_{3}$ were blinded		
Granstrom, 1995 Sweden	RCT	N=181	G$_{1}$:IOL at 12h	7/13 (53.8%)	Intermediate
			G$_{2}$:IOL at 24h		
Moberger, 1997 Sweden	Quasi-RCT	N=380	G$_{1}$: IOL at 12h.	3/13 (23.1%)	Low
			G$_{2}$: IOL at 24h.		
Akyol, 1999 Turkey	RCT	N=126	G1: Immediate IOL with IV oxytocin	7/13 (53.8%)	Intermediate
			G2: Expectant up to 24h followed by IOL (IV oxytocin)		
Ayaz, 2008 Saudi Arabia & Pakistan	Quasi RCT	N=84	G1: Immediate IOL (oral misoprostol)	5/13 (38.5%)	Low
			G2: Expectant up to 24h		
Bashir, 2017 Pakistan	Quasi-experimental	N=120	G$_{1}$: Immediate IOL (PGE2 or oxytocin)	6/13 (46.2%)	Intermediate
			G$_{2}$: Expectant up to 24h		
Chung, 1992 Hong Kong	RCT	N=59	G1: Immediate IOL with PGE$_{2}$ (during the first 12h)	6/13 (46.2%)	Intermediate
			G2: Expectant up to 24h (placebo KY jelly)		
Da Graca Krupa, 2005 Brazil	RCT	N=150	G$_{1}$: Immediate IOL (misoprostol) followed by IV oxytocin	7/13 (53.8%)	Intermediate
			G$_{2}$: Expectant up to 24h followed by IOL with IV oxytocin		
Fatima, 2015 Pakistan	RCT	N=200	G$_{1}$: Immediate IOL with PGE1 (Misoprostol)	6/13 (46.2%)	Intermediate
			G$_{2}$: Expectant up to 24h		
Grant, 1992 UK	RCT	N=444	G$_{1}$: Immediate IOL with oxytocin	9/13 (69.2%)	Intermediate
			G$_{2}$: Expectant up to the following morning (9–33h) followed by IV oxytocin if needed		
Javaid, 2008 Pakistan	RCT	N=100	G$_{1}$:Immediate IOL (oral misoprostol)	2/13 (15.4%)	Low
			G$_{2}$: Expectant up to 24h		
Mahmood, 1995 Scotland (UK)	RCT	N=100	G$_{1}$: Immediate IOL with PGE2	5/13 (38.5%)	Low
			G$_{2}$: Expectant up to 24h.		
Maqbool, 2014 Pakistan	RCT	N=560	G$_{1}$: Immediate IOL with PGE1 (misoprostol)	4/13 (30.7%)	Low
			G$_{1}$: Expectant up to 24h		
Shetty, 2002 UK	RCT	N=61	G$_{1}$: Immediate IOL with oral misoprostol	7/13 (53.8%)	Intermediate
			G$_{2}$: Expectant up to 24h Followed by Prostaglandins or IV oxytocin depending on Bishop score		
Wagner, 1989 USA	Quasi–RCT	N=182	G$_{1}$: Immediate IOL with oxytocin	5/13 (38.5%)	Low
			G$_{2}$: Expectant up to 24h.		
Natale, 1994 Canada	RCT	N=262	G$_{1}$: IOL at 8h since SROM	8/13 (61.5%)	Intermediate
			G$_{2}$: Expectant up to 48h		
Ottervanger, 1996 The Netherlands	RCT	N=123	G$_{1}$: Immediate IOL with oxytocin	8/13 (61.5%)	Intermediate
			G$_{2}$: Expectant up to 48h		
Van der Walt, 1989 South Africa	Quasi–RCT	N=60	G$_{1}$: Immediate IOL with IV oxytocin	3/13 (23.1%)	Low
			G$_{2}$: Immediate IOL with PGE$_{2}$		
			G$_{3}$: Expectant up to 48h.		
Hannah, (1996)) TERPROM 9 published papers	RCT	N=5,041	G$_{1}$: Immediate IOL with IV oxytocin	9/13 (69.2%)	Intermediate
			G$_{2}$: Immediate IOL with prostaglandins		
			G$_{3}$: EM up to 96h folllowed by IOL (IV oxytocin)		
			G4: EM up to 96h followed by IOL (prostaglandins)		
Rydhstrom, 1991 Sweden	RCT	N=369	G$_{1}$: Immediate IOL with oxytocin	5/13 (38.5%)	Low
			G$_{2}$:Expectant up to 80h.		
Yasmin, 2013 Pakistan	Quasi-experimental	N=100	G$_{1}$: Immediate IOL with PGE$_{2}$	5/13 (38.5%)	Low
			G$_{2}$: Expectant up to 72h.		
Alcalay (1996) Israel	Quasi RCT	N=154	G1: Immediate IOL with IV oxytocin	7/13 (53.8%)	Intermediate
			G2: EM (no limit)		
Duff, 1984 USA	Quasi-RCT	N=134	G$_{1}$: IOL by 12h with IV Oxytocin	7/13 (53.8%)	Intermediate
			G$_{2}$: EM with no time limit		
McCaul, 1997 USA	RCT	N=96	G$_{1}$: Expectant (Unclear how long)	4/13 (30.7%)	Low
			G$_{2}$:IOL with Oxytocin at least 4h after SROM		
			G$_{3}$:IOL with PGE2 at least 4h after SROM		
Morales, 1986 USA	Quasi-RCT	N=317	G$_{1}$:Immediate IOL with oxytocin	5/13 (38.5%)	Low
			G$_{2}$:Expectant (No time limit)		
Tamsen, 1990 Sweden	RCT	N=93	G$_{1}$:Immediate IOL with oxytocin	6/13 (46.2%)	Intermediate
			G$_{2}$:Expectant (no time limit)		
Ezra (2004) Israel	Observational case-control	N=411	G1: Cases of PROM with chorioamnionitis or neonatal infection	8/12 (66.6%)	Intermediate
			G2 Control: Cases of PROM with no chorioamnionitis/neonatal infection		
Paraiso, 2013 Spain	Observational Retrospective	N=115	G$_{1}$:Immediate IOL with oxytocin	1/12 (8.3%)	Low
			G$_{2}$:Expectant up to 24h.		
Sadeh-Mestechkin,2016 Israel	Observational Retrospective	N=325	G$_{1}$: Immediate IOL	9/12 (75%)	Intermediate
			G$_{2}$: Expectant up to 48h.		
Shalev, 1995 Israel	Observational study Prospective	N=566	G$_{1}$: IOL at 12h. with IV oxytocin	7/12 (58.3%)	Intermediate
			G$_{2}$:IOL at 72h. followed by oxytocin		
Zamzami, 2006 Saudi Arabia	Observational case-control	N=344	G$_{S}$: Divided in 2 groups chosen by Dr.	4/12 (33.3%)	Low
			G$_{S1}$:Immediate IOL with oxytocin		
			G$_{S2}$:Expectant up to 24h		
			G$_{C}$:Women in spontanous labour with intact membranes		

Quality Categories: Low <40%; Intermediate 41% -80%; High >81%

## Discussion

The search strategy for this systematic review did not identify any prospective studies that answered the question of whether expectant management and a reduced number of vaginal examinations are associated with a higher rate of normal birth and a lower rate of chorioamnionitis.

Although some studies looked at caesarean section, they did not provide information about physiological labour and birth. Systematic reviews of what matters to women around the world indicate that “normal/physiological birth” is valued by most [30], and the recent Lancet Series on reducing caesarian section noted that one effective way of doing this is to increase physiological labour and birth [31].

Through the process of searching and gathering studies, it also became evident that there are no studies on this topic that looked at normal birth as an outcome, most of the studies looked at reducing caesarean section as opposed to increase physiological birth. The Lancet midwifery series supports more studies with physiological birth as an outcome.

The studies found identified that the management of prelabour rupture of membranes is a matter of global interest, as there were studies published in the five continents, both in developed and high income countries, as well as in developing countries. Apart from the fact that the use of epidural was reported more often in Europe than other parts of the world, there were no particular trends depending on the country, making the findings more generalisable.

The following limitations of this systematic review were identified; This systematic review was not registered, most of the studies found were of poor quality, very few had computerised randomisation, the primary and secondary outcomes were not stated or these were not clear, the diagnosis of chorioamnionitis or neonatal infection was not blinded, the diagnosis of chorioamnionitis and neonatal infection varied a lot and in some cases the definitions were not appropriate, making it very difficult to perform a meta-analysis due to the high degree of heterogeneity.

On the other hand, most of the studies were published more than ten years ago, and although the issue of prelabour rupture of membranes has not changed, and women continue to break their waters before going into labour, practices around birth have changed.The secondary analysis undertaken for this review on the induction agent used in the TERMPROM study, which is the largest study to date, published by Hannah et al. [27] revealed that there was no statistical difference in the risk of chorioamnionitis when labour was induced with prostaglandins followed by IV oxytocin if needed in comparison to when labour was induced with IV Oxytocin on its own. Given the more widespread use of prostaglandins for labour induction in recent years, there is now a need for updated studies taking this change of practice into account.

The lack of attention to the impact of frequency of vaginal examinations is also a concern, both from the potential impact on infection (and the consequent potential need for antibiotics, in light of the increasing antibiotic resistance) and because vaginal examinations can often cause anxiety to woman [32]. A study published in Sweden, showed that 45% of women found the gynaecological examination to be a “negative” experience [33]. Therefore, it is important to minimise the number of vaginal examinations performed during their labours.

## Conclusion

There are no published studies (RCTs or observational) that have looked at expectant management and an approach to minimise vaginal examinations during labour for prelabour rupture of membranes to maximise the chances of physiological birth and minimise chorioamnionitis. Considering that vaginal examinations are a routine intervention during most labours and that there is evidence that vaginal examinations are one of the strongest correlators of chorioamnionitis, it is crucial to carry out more studies that find ways to monitor the progress of labour using other means. Future studies in the management of prelabour rupture of membranes should be designed and powered to include both physiological birth and chorioamnionitis as birth outcomes.

### Supplementary Information

**Additional file 1: Supplementary material.** This document contains all the studies from this review, it is an extended version of Table 4, and it has several tables with all the studies included in the review organised by the length of expectant management.

## Data Availability

Supplementary file contains all the studies found in this systematic review.
