# Supplementary material for: Systematic review on the management of term prelabour rupture of membranes
Source: BMC Pregnancy Childbirth. 2023 Sep 8;23:650. doi: 10.1186/s12884-023-05878-x (PMC10492345; doi:10.1186/s12884-023-05878-x)
Supplement: Supplementary file 1 — Additional file 1: Supplementary material. This document contains all the studies from this review, it is an extended version of Table 4, and it has several tables with all the studies included in the review organised by the length of expectant management. [file 12884_2023_5878_MOESM1_ESM.pdf]

Supplementary material to “Systematic review on the management of term prelabour rupture of membranes”

Supplementary table 1: RCT studies comparing Immediate IOL vs EM up to 12h.

**First author, year Country**	**Type of study**	**Total N of participants n per group**	**Intervention & comparison**	**Outcomes**	**Results**	**Quality**
Poornima, 2011<br>India	RCT<br>Doesn't state randomisation method	N=100<br>n_1=50<br>n_2=50	G_1: Immediate IOL with PGE_2<br>(Max 2 doses/6h apart)<br>G_2:Expectant up to 12h. Followed by IV oxytocin if needed	Primary & secondary not stated<br>Outcomes:<br>Length of SROM<br>Length of time between SROM and cervical dilatation=3cm<br>Length of active labour<br>Clinical chorioamnionitis<br>clinical neonatal infection<br>Definitions for chorioamnionitis and neonatal infection not given<br>Number of VEs NOT reported	More Spontaneous labours, more vaginal births, less CS in EM.<br>No significant differences in infections.<br>CS:<br>G_1=40% G_2=27%<br>Clinical chorioamnionitis:<br>G_1=0 G_2=0<br>Clinical neonatal infection:<br>G_1=2/50(4%) G_2=2/50(4%)	5/13<br>(38.5%)
Ray, 1992<br>USA	RCT<br>Randomisation list mantained by pharmacy staff	N=140<br>n_1=40<br>n_2=55<br>n_3=45	G_1: Immediate IOL with PGE_2<br>(max 2 doses/6h apart)<br>G_2: Immediate IOL with oxytocin<br>G_3: Expectant up to 12h<br>G_1 & G_3 were blinded	Primary & secondary not stated<br>Outcomes:<br>Length of SROM<br>CS, Chorioamnionitis and neonatal sepsis<br>Number of VEs not reported	No SSD between the 3 groups in CS<br>No cases of neonatal infection<br>Shorter length of SROM with PGE_2 than IV oxytocin or placebo<br>definition of chorioamnionitis only temperature $\geq 38°$C reported<br>maternal infection instead of chorioamnionitis<br>Results provided only % not absolute numbers.<br>CS:<br>Nulliparous:<br>G_1= 14.5% G_2=20.7% G_3=19%<br>Multiparous:<br>G_1=0 G_2=15.3% G_3=12.5%<br>Maternal infection:<br>Nulliparous:<br>G_1=9.5% G_2=34.5% G_3=33.3%<br>Multuparous:<br>G_1=0 G_2=11.5% G_3=8.3%	5/13<br>(38.5%)

Supplementary table 2: RCT studies comparing IOL at 12h vs EM up to 24h.

First author, year<br>Country	Type of study	Total N<br>of participants<br>n per group	Intervention<br>& comparison	Outcomes	Results	Quality
Granstrom, 1995<br>Sweden)	RCT<br>allocation by drawing<br>a sealed<br>& numbered envelope	N=181<br>n_1=91<br>n_2=90	G_1:IOL at 12h<br>G_2:IOL at 24h	Primary & secondary not stated<br>Outcomes: Length of SROM<br>Length of labour<br>CS<br>Number of VEs NOT reported	EM resulted in higher incidence of<br>spontaneous ripening of the cervix<br>combined with spontaneous onset of labour<br>and spontaneous vaginal delivery<br>maternal and neonatal complications<br>were similar in both groups<br>No significant differences in:<br>length of SROM<br>length of labour<br>or instrumental births.<br>More CS in G_1<br>CS: G_1:11/91(12%) G_2:7/90(8%)<br>No definition of chorioamnionitis,<br>suspected infection, or neonatal infection<br>Swabs were taken from all babies<br>with or without symptoms of infection<br>Positive bacterial cultures in neonates<br>were similar in both groups:<br>G_1:30/91(33%) vs G_2:26/90(29%)	7/13<br>(53.8%)
Moberger, 1997<br>Sweden	Quasi-RCT<br>Allocated at random<br>doesn't say how<br>according to the cervix<br>(favourable/unfavourable)<br>Unclear if stratification by<br>cervical ripeneness	N=380<br>n_1=Doesn't say<br>n_2=Doesn't say	G_1: IOL at 12h.<br>G_2: IOL at 24h.	Primary: Neonatal outcome<br>other: CS<br>Number of VEs NOT reported	Perinatal mortality=0<br>No SSD in admissions to NICU<br>spontaneous births, inductions,<br>instrumentals, length of labour<br>antibiotics in the mother<br>Chorioamnionitis not defined or reported<br>only signs of infection in the mother,<br>doesn't say when or which signs<br>Neonatal infection not clearly reported,<br>suspicious and definitive neonatal infection<br>reported together<br>Neonatal infection: G_1=9 cases G_2=6 cases<br>CS:G_1=8% G_2=6%	3/13<br>(23.1%)

Supplementary table 3: RCT studies comparing Immediate IOL vs EM up to 33h

**First author, year**<br>**Country**	**Type of study**	**Total N of participants**<br>**n per group**	**Intervention & comparison**	**Outcomes**	**Results**	**Quality**
Akyol, 1999<br>Turkey	RCT	N=126<br>(underpowered)<br>n_1=52<br>n_2=74	G1: Inmediate IOL<br>with IV oxytocin<br>G2: Expectant up to 24h<br>followed by IOL with IV oxytocin<br>Participants in EM were offered<br>an elective CS if necessary	Primary: Definitive or probable<br>neonatal infection<br>Secondary:CS<br>Reports number of VEs	Selective reporting:<br>It does not mention cases of<br>probable or definitive<br>neonatal infection<br>CS:<br>G1(AM)=10/52(19.2%)<br>G2(EM)=21/74 (28.4%)	7/13<br>(53.8%)
Ayaz, 2008<br>Saudi Arabia<br>& Pakistan	Quasi RCT<br>Participants chose<br>1 of 2 cards<br>labelled as S or C<br>S=Study<br>C=Conservative	N=84<br>n_1=42<br>n_2=42	G1: Immediate IOL<br>with oral misoprostol<br>(every 4h, max 4 doses)<br>G2: Expectant up to 24h	Does not specify<br>primary or secondary outcomes<br>Reports:<br>Interval from SROM<br>to onset of labour<br>interval from SROM<br>to delivery<br>CS and vaginal delivery<br>maternal & neonatal complications<br>No definition for:<br>neonatal sepsis or<br>chorioamnionitis<br>No mention of N of VE	The interval between<br>SROM and onset of labour<br>and between SROM and<br>delivery was significantly<br>shorter in the study group<br>Higher rate of CS in EM<br>Women were offered a CS<br>if not in labour<br>by 24h of EM	5/13<br>(38.5%)
Bashir, 2017<br>Pakistan	Quasi-experimental<br>allocation by<br>randomisation	N=120<br>n_1=60<br>n_2=60	G_1: Immediate IOL<br>(PGE2 or oxytocin)<br>G_2: Expectant up to 24h	Primary/secondary not stated<br>Outcomes:<br>Mode of delivery<br>Neonatal infection<br>Fetal dystress<br>NICU admission $\geq$24h.<br>Post-partum pyrexia<br>Endometritis<br>Mean Hospital stay	No significant difference in:<br>Mode of delivery<br>Neonatal infection<br>Endometritis<br>hospital stay<br>endometritis<br>more postpartum fever in EM<br>Number of VEs NOT reported	6/13<br>(46.2%)
Chung, 1992<br>Hong Kong	RCT<br>Allocated by<br>computerized<br>set of random<br>numbers	N=59<br>n_1=30<br>n_2=29	G1: Inmmediate IOL<br>(during the first 12h)<br>with PGE2<br>G2: Expectant up to 24h<br>with placebo (KY jelly)	Length of SROM<br>at onset of labour and<br>at delivery<br>Length of<br>labour<br>CS rate<br>No mention of<br>Number of VEs	Women who received PGE2<br>went into labour sooner<br>and gave birth earlier.<br>1 case of uterine rupture in G1<br>Chorioamnionitis not reported<br>No significant differences in:<br>duration of labour<br>operative delivery<br>There were no cases of<br>neonatal infection	6/13<br>(46.2%)

Supplementary table 3: RCT studies comparing Immediate IOL vs EM up to 33h

First author, year<br>Country	Type of study	Total N of participants<br>n per group	Intervention & comparison	Outcomes	Results	Quality
Da Graca Krupa,<br>2005<br>Brazil	RCT<br>Randomised by<br>envelopes	N=150<br>n_1=75<br>n_2=75	G_1: Inmediate IOL<br>with misoprostol (PGE1)<br>max 4 doses of 25mg at 6h intervals<br>followed by IV oxytocin<br>if needed<br>G_2: Expectant up to 24h<br>followed by IOL with IV oxytocin<br>if needed	Primary: Time from recruitment<br>to delivery<br>Secondary:<br>Latency period<br>length of hospitalization<br>CS rate<br>contractility pattern<br>labour and delivery complications<br>maternal & neonatal morbidity<br>Chorioamnionitis was not reported<br>No mention of<br>Number of VEs	Latency period and<br>time from recruitment to birth<br>was shorter in G1<br>More hypercontractility<br>and tachysystole in G1<br>No significant differences in CS<br>or Apgar scores<br>No cases of neonatal infection<br>or admissions to NICU reported)	7/13<br>(53.8%)
Fatima, 2015<br>Pakistan	RCT<br>Lottery method	N=200<br>n_1=100<br>n_2=100	G_1: Immediate IOL with PGE1<br>(Misoprostol)<br>G_2: Expectant up to 24h	Primary/secondary not stated<br>Outcomes:<br>IOL to labour interval<br>Length of labour<br>Mode of delivery<br>Apgars at 5min<br>NICU admission	Shorter<br>IOL to labour interval in G1<br>Maternal fever:<br>G2 (10%) vs G1(3.33%<br>No significant diferences in:<br>Length of labour<br>Modeo of birth<br>admissions to NICU<br>Apgar score<br>Neonatal complications<br>Number of VEs NOT reported)	6/13<br>(46.2%)
Grant, 1992<br>UK	RCT<br>Opaque & sealed<br>envelopes	N=444<br>n_1=219<br>n_2=225	G_1: Immediate IOL with oxytocin<br>G_2: Expectant up to the following<br>morning (9–33h)<br>followed by IV oxytocin if needed	Primary: CS<br>Other:<br>Length of latent phase<br>Length of active labour<br>Analgesia<br>VEs<br>Maternal pyrexia<br>($T \geq 37.1°C$) in ≥1 occassions<br>neonatal infection	Fewer women in G_2 had ≥4VEs<br>Neonatal infection<br>assessed by neonatologist<br>not aware of allocation,<br>but definition not given.<br>Chorioamnionitis not reported.<br>CS:<br>G1:38/219(17%) G2:25/225(11%)<br>SVD:<br>G1:113/219(52%) G2:141/225(63%)<br>VEs: (continuous)<br>M(SD): G1:2.84(1.36) G2:3.37(1.42)<br>Neonatal infection:<br>G1:0 G2:1/225(0.4%)	9/13<br>(69.2%)

Supplementary table 3: RCT studies comparing Immediate IOL vs EM up to 33h

**First author, year Country**	**Type of study**	**Total N of participants n per group**	**Intervention & comparison**	**Outcomes**	**Results**	**Quality**
Javaid, 2008<br>Pakistan	RCT<br>Does not mention<br>how randomisation or<br>allocation took place	N=100<br>n_1=50<br>n_2=50	G_1:Immediate IOL (oral misoprostol)<br>G_2: Expectant up to 24h	Primary outcome not stated<br>only mentions<br>"maternal & fetal outcomes"<br>It looks at:<br>CS<br>Length of hospital stay<br>need for augmentation<br>clinical chorioamnionitis<br>(not defined)<br>Postpartum fever<br>Infected wound after CS<br>PPH<br>Neonatal morbidity<br>(not defined)<br>Admission to NNU	No tables provided<br>No absolute numbers provided<br>Only percentages<br>or higher/lower statements<br>but not specific numbers<br>Chorioamnionitis:<br>G_1:3% G_2:7.8%<br>CS: G_1:24% G_2:34%	2/13<br>(15.4%)
Mahmood, 1995<br>Scotland (UK)	RCT<br>Randomised list<br>& sealed envelopes	N=100<br>n_1=50<br>n_2=50	G_1: Immediate IOL with PGE2<br>(1mg/dose, 6h apart, max 2doses)<br>G_2: Expectant up to 24h.	Primary & secondary not stated<br>Outcomes:<br>Length of SROM<br>need for IV oxytocin<br>need for analgesia<br>Number of VEs NOT reported<br>chorioamnionitis NOT reported<br>No definition of neonatal infection	Time from SROM–onset of labour<br>& total length of SROM<br>longer in EM<br>No significant differences in:<br>CS, need for IV oxytocin<br>meconium<br>intrapartum pyrexia<br>length of 2nd stage<br>PPH, apgars and IV ATB for baby	5/13<br>(38.5%)
Maqbool, 2014<br>Pakistan	RCT<br>allocated by<br>"lottery method"	N=560<br>n_1=280<br>n_2=280	G_1: Immediate IOL<br>with PGE1 (misoprostol)<br>100 µg up to 5 doses/4h apart<br>G_2 :Expectant up to 24h	Primary & secondary not stated<br>Outcomes: Type of birth<br>chorioamnionitis<br>No definition for chorioamnionitis<br>Number of VEs not reported	More CS<br>and chorioamnionitis in EM	4/13<br>(30.7%)

Supplementary table 3: RCT studies comparing Immediate IOL vs EM up to 33h

First author, year<br>Country	Type of study	Total N of participants<br>n per group	Intervention & comparison	Outcomes	Results	Quality
Shetty, 2002<br>UK	RCT<br>(Sealed envelopes)	N=61<br>n_1=30<br>n_2=31	G_1: Immediate IOL<br>with oral misoprostol<br>G_2: Expectant up to 24h<br>Followed by Prostaglandins or<br>IV oxytocin<br>depending on Bishop score	Primary: Number of women in active<br>labour within 24h<br>since rupture of membranes<br>Assess patient's preference<br>for management<br>Secondary:Total length of SROM<br>(from SROM till Birth)<br>Spontaneous vaginal delivery<br>Instrumental<br>CS<br>Number of VEs<br>Maternal Pyrexia<br>Chorioamniniotitis not defined<br>Chorioamnionitis not reported<br>Admission to NNU<br>Neonatal infection not defined<br>Neonatal infection not reported	More active labour within 24h in G_1<br>G_1:28/30 (93.3%) G_2:17/31(54.8%)<br>No significant difference in:<br>satisfaction<br>maternal or neonatal outcomes<br>Number of VEs	7/13<br>(53.8%)
Wagner, 1989<br>USA	Quasi–RCT<br>Randomised by<br>last digit in<br>participants' medical<br>records<br>even number: EM<br>odd number: AM	N=182<br>n_1=86<br>n_2=96	G_1: Immediate IOL with oxytocin<br>G_2: Expectant up to 24h.	Doesn't state<br>primary/secondary ouctomes<br>Outcomes:<br>Length of SROM<br>CS<br>Intra–amniotic infection<br>neonatal infection<br>endometritis<br>Number of VEs reported	No significant differences in CS,<br>neonatal infection<br>& endometritis<br>No cases of clinical<br>intra-amniotic infection<br>All placentas had histologic exam<br>25% showed inflammatory signs<br>(Histologic chorioamnionitis)<br>None symptomatic<br>All neonatal infections<br>ocurred in those who<br>had a VE on enrolment<br>in the study<br>CS:<br>G1=12/86(14%) G2=15/96(15.6%)<br>Neonatal infection:<br>G1=0 G2=5/96(5.2%)	5/13<br>(38.5%)

Supplementary table 4: RCT studies comparing Immediate IOL vs EM up to 48h.

First author, year<br>Country	Type of study	Total N of participants<br>n per group	Intervention & comparison	Outcomes	Results	Quality
Natale, 1994<br>Canada	RCT<br>Does not mention how randomisation took place	N=262<br>(Underpowered)<br>n_1=129<br>n_2=133	G_1: IOL at 8h since SROM<br>G_2: Expectant up to 48h	Primary: CS<br>Secondary:<br>Clinical neonatal-maternal infection<br>Number of VEs NOT reported	No SSD in CS<br>More spontaneous labours in EM<br>Neonatal sepsis not defined<br>Neonatal sepsis not reported<br>Only reported admissions to NICU<br>Reported antibiotics given—not necessarily related to treatment but to hospitals' protocols<br>Chorioamnionitis not defined<br>only reported findings from histological exam of placentas not how many participants had clinical signs of infection<br>CS: G_1=11.2% G_2=13.1%<br>Histologic chorioamnionitis:<br>G_1=20.2% G_2=33.3%	8/13<br>(61.5%)
Ottervanger, 1996<br>The Netherlands	RCT<br>randomisation by sealed & opaque envelopes)	N=123<br>n_1=61<br>n_2=62	G_1: Immediate IOL with oxytocin<br>G_2: Expectant up to 48h	Primary: CS<br>Secondary: Instrumental births<br>use of analgesia<br>maternal infectious morbidity<br>neonatal infectious morbidity<br>neonatal re–admission<br>Number of VEs NOT reported<br>Powered to CS<br>stopped earlier<br>interim analysis revealed significant differences in CS	More spontaneous labours in EM<br>More CS and Instrumentals in AM<br>Similar length of labour in both groups<br>Chorioamnionitis not defined<br>Only reported maternal infectious morbidity<br>Not clear if that relates to chorioamnionitis endometritis or both<br>Neonatal infection not defined<br>No cases of neonatal infection<br>All participants had cervical cultures and all babies had gastric aspirates' cultures and section of the cord was sent to histopathology<br>High rates of positive cultures and histological examination<br>Low numbers of clinical signs of infection in women or babies<br>CS: G_1=4/61(6.6%) G_2=2/62 (3.2%)<br>Maternal infectious morbidity:<br>G_1=1/61(1.6%) G_2=2/62(3.2%)<br>neonatal infectious morbidity:<br>G_1=0 G_2=0	8/13<br>(61.5%)

Supplementary table 4: RCT studies comparing Immediate IOL vs EM up to 48h.

First author, year<br>Country	Type of study	Total N<br>of participants<br>n per group	Intervention & comparison	Outcomes	Results	Quality
Van der Walt, 1989<br>South Africa	Quasi–RCT<br>(allocated according<br>to a numerical list<br>kept on Labour<br>ward)	N=60<br>n_1=20<br>n_2=20<br>n_3=20	G_1: Immediate IOL with<br>IV oxytocin<br>G_2: Immediate IOL with PGE_2<br>G_3: Expectant up to 48h.	Does not state<br>primary or secondary outcomes<br>Outcome of labour<br>maternal<br>& neonatal welfare<br>Time between<br>SROM and active labour (3cm)<br>Length of labour (3cm–delivery)<br>CS<br>Endometritis<br>defines chorioamnionitis<br>but does not report it<br>Number of VEs NOT reported	In G_3(EM):90% had spontaneous labour<br>No cases of CS<br>Active labour was<br>shorter in G_3 than G_1 and G_2<br>EM and AM with PGE_2 more effective than<br>AM with IV oxytocin<br>No CS in G_2 and G_3<br>No significant maternal morbidity<br>1 case of neonatal positive blood cultures<br>but does not say which group<br>Bias: Failed IOL defined as<br>no spontaneous labour within 12h in G_1<br>whilst participants in G_2 were given 18h<br>(3 doses/6h apart)<br>No cases of neonatal death<br>No cases of endometritis<br>EM not led to higher infection rate<br>CS: G1:6/20(30%) G2=0 G3=0<br>Neonatal sepsis:<br>G1: 1/20 (5%) G2=0 G3=0	3/13<br>(23.1%)

Supplementary table 5: RCT studies comparing Immediate IOL vs EM up to 96h.

First author, year<br>Country	Type of study	Total N<br>of participants<br>n per group	Intervention & comparison	Outcomes	Results	Quality
Hannah, (1996))<br>TERMPROM<br>International<br>multicentre<br>9 published papers	RCT	N=5,041<br>n_1=1,258<br>n_2=1,259<br>n_3=1,263<br>n_4=1,261	G_1: Immediate IOL<br>with IV oxytocin<br>G_2: Immediate IOL<br>with prostaglandins<br>G_3: EM up to 96h<br>folllowed by IOL<br>with IV oxytocin<br>G4: EM up to 96h<br>followed by IOL with<br>prostaglandins if needed	Primary: Neonatal infection<br>Secondary: CS +<br>Women's views<br>Number of VEs were reported	No significant differences for:<br>neonatal infection<br>CS<br>and chorioamnionitis<br>(when using prostaglandins)	9/13<br>(69.2%)
Rydhstrom, 1991<br>Sweden	RCT<br>(Sealed envelopes)	N=369<br>(under powered)<br>n_1=139<br>n_2=138	G_1: Immediate IOL with<br>oxytocin<br>G_2:Expectant up to 80h.	Primary:obstetric intervention:<br>composite of CS or Instrumental<br>birth and short term neonatal<br>morbidity<br>Other: Length of SROM<br>fever, antibiotics<br>neonatal morbidity:<br>Pneumonia, sepsis<br>meningitis.<br>Number of VEs not reported<br>Power calculation based on<br>reducing rate of obstetric<br>intervention<br>and abnormal CTG<br>by 50% (N=700)<br>Stopped sooner because<br>interim analysis revealed<br>hypothesis could not be tested	No statistical differences in:<br>CS, instrumentals, CTG traces.<br>No definition of abnormal CTG trace.<br>Chorioamnionitis not reported.<br>Only intrapartum fever<br>$\geq$ 38°C during 1st stage of labour,<br>more cases of fever in those with epidural.<br>No SSD in endometritis.<br>More neonatal infectius morbidity in G_2,<br>but doesn't say exactly what,<br>as it is a composite could be:<br>pneumonia, sepsis, meningitis pemphigus or<br>impetigo<br>does not define sepsis.<br>CS:<br>G_1=4/139(2.9%) G_2=5/138(3.6%)<br>Neonatal infection:<br>G_1=1/139 G_2=6/138(4.3%)	5/13<br>(38.5%)

Supplementary table 5: RCT studies comparing Immediate IOL vs EM up to 96h.

**First author, year Country**	**Type of study**	**Total N of participants n per group**	**Intervention & comparison**	**Outcomes**	**Results**	**Quality**
Yasmin, 2013 Pakistan	Quasi-experimental Participants could choose allocation group if they didn't want to the clinician chose 87% were unable to decide	N=100 n_1=50 n_2=50	G_1: Immediate IOL with PGE_2 G_2: Expectant up to 72h	Primary & secondary not stated maternal & fetal complications length of hospital stay mode of birth costs mentions number of VEs less in EM but not significantly different but doesn't report figures	No definition for SVD. More SVD in EM, less CS in EM. No significant differences in: CS, Instrumentals, neonatal sepsis Chorioamnionitis not reported. Although it says 1st cause of CS was chorioamnionitis. Only mild intrapartum fever reported, doesn't give exact figures and doesn't specify what mild fever is. More mild intrapartum fever in EM. Doesn't report results on epidurals. Costs were higher in AM SVD: G1=30/50(60%) G2=40/50(80%) $p<0.05$ CS: G1=11/50(22%) G2=6/50(12%) p=0.28 Neonatal sepsis: G1=1/50(2%) G2=1/50(2%)	5/13 (38.5%)

Supplementary table 6: RCT studies comparing Immediate IOL vs EM with no time limit

**First author, year Country**	**Type of study**	**Total N of participants n per group**	**Intervention & comparison**	**Outcomes**	**Results**	**Quality**
Alcalay (1996)<br>Israel	Quasi RCT<br>Randomised by<br>table of random<br>numbers	N=154<br>n_1=74<br>n_2=80	G1: Immediate IOL<br>with IV oxytocin<br>G2: EM (no limit)	Doesn't specify<br>primary or secondary outcomes<br>Reports:<br>SVD, CS, Number of VEs,<br>Neonatal infection<br>and Chorioamnionitis	More SVD in EM<br>EM is safe<br>No statistically significant<br>differences in<br>CS, neonatal infection<br>or chorioamnionitis	7/13<br>(53.8%)
Duff, 1984<br>USA	Quasi-RCT<br>Group allocation<br>based on<br>the day of the week<br>of patien's admission<br>to hospital	N=134<br>n_1=59<br>n_2=75	G_1: IOL by 12h with IV Oxytocin<br>G_2: EM with no time limit	Primary/secondary not stated<br>Outcomes:<br>Length of labour<br>Length of SROM<br>CS<br>Intra-amniotic infection<br>Endometritis<br>proven neonatal sepsis<br>Number of VEs NOT reported	G_1had more intra-amniotic infection,<br>more CS and longer labours.<br>CS:<br>G1=12/59(20%) G2=6/75(8%) $p<0.005$<br>Intra-amniotic infection:<br>G1=10/59 (17%) G2=3/75(4%) $p<0.005$<br>Proven neonatal sepsis:<br>G1=1/59(1.7%) G2=0	7/13<br>(53.8%)
McCaul, 1997<br>USA	RCT<br>Computer generated<br>random group<br>allocation	N=96<br>n_1=36<br>n_2=25<br>n_3=35	G_1: Expectant (Unclear how long)<br>G_2:IOL with Oxytocin<br>at least 4h after SROM<br>G_3:IOL with PGE2<br>at least 4h after SROM	Primary & secondary not stated<br>Outcomes: CS, Length of SROM<br>Length of labour<br>Length of hospital stay<br>VEs were analysed in G_1 and G_2<br>but not in G_3<br>selective reporting	No significant differences in CS,<br>length of labour, neonatal morbidity<br>or neonatal stay<br>No infant required ventilation<br>or treatment for sepsis<br>Longer length of SROM<br>and length of maternal stay in EM<br>than in AM groups<br>neonatal sepsis not defined	4/13<br>(30.7%)
Morales, 1986<br>USA	Quasi-RCT<br>Allocated to AM or EM<br>according to day of the week<br>of admission and<br>the hospital case number	N=317<br>n_1=150<br>n_2=167	G_1:Immediate IOL with oxytocin<br>G_2:Expectant (No time limit)	Primary & secondary not stated<br>Looks at:<br>Intra-amniotic infection<br>Failed IOL<br>Failure to progress<br>CS<br>Neonatal infection<br>Number of VEs NOT reported	No neonatal infections<br>Less intra-amniotic infection in EM<br>Less CS in EM<br>No difference in length of hospitalization<br>CS:<br>G_1=31/150(21%) G_2=11/167(7%)<br>Intra-amniotic infection:<br>G_1=18/150(12%) G_2=7/167(4%)	5/13<br>(38.5%)

Supplementary table 6: RCT studies comparing Immediate IOL vs EM with no time limit

**First author, year**<br>**Country**	**Type of study**	**Total N of participants n per group**	**Intervention & comparison**	**Outcomes**	**Results**	**Quality**
Tamsen, 1990<br>Sweden	RCT<br>Does not mention how randomization took place	N=93<br>n_1=43<br>n_2=50	G_1:Immediate IOL with oxytocin<br>G_2:Expectant (no time limit)	Primary & secondary not stated<br>Looks at:<br>Time from SROM to active labour<br>Time from active labour to birth<br>Time from SROM to birth<br>Results subdivided by parity<br>Need for augmentation<br>SVD<br>Vacuum extraction<br>CS<br>Admission to NNU >7days<br>Maternal infection not defined<br>Neonatal infection not defined	Shorter time from SROM to birth in AM for both Primiparas & multiparas<br>SVD:<br>G_1=40/43(93%) G_2=40/50(80%)<br>CS:<br>G_1=0 G_2=4/50(8%)<br>Maternal infection:<br>G_1=0 G_2=1/50(2%)<br>Neonatal infection:<br>G_1=0 G_2=2/50(4%)	6/13<br>(46.2%)

Supplementary table 7: Observational studies (n=5)

**First author, year**<br>**Country**	**Type of study**	**Total N of participants n per group**	**Intervention & comparison**	**Outcomes**	**Results**	**Quality**
Ezra (2004)<br>Israel	Observational:<br>retrospective<br>case-control	N=411<br>n_1=132 (cases)<br>n_2=279 (control)	G1: Cases of PROM with<br>clinical chorioamnionitis<br>or neonatal infection<br>Bias: since either maternal<br>or neonatal infection would clasify<br>to be a case was difficult to analyse<br>which treatment<br>would be best for chorioamnionitis<br>G2 Control: Cases of PROM<br>with no evidence of<br>chorioamnionitis<br>or neonatal infection.<br>Chorioamnionitis def:<br>at least 2 of the following:<br>Maternal temperature > 37.8°C<br>Maternal white cell count > 15,000<br>Foul smelling liquor<br>Maternal tachycardia >100bpm<br>Fetal tachycardia >160bpm<br>Uterine tenderness	Main outcome measures:<br>Clinical chorioamnionitis<br>and neonatal infection.<br>2º: C/S, instrumental<br>It compared<br>3 management options:<br>1) Immediate IOL<br>2)Expectant up to 24h<br>3) Expectant over 24h<br>With 3 comparisons<br>EM>24h vs EM up to 24h<br>EM>24h vs Immediate IOL<br>EM up to 24h vs Immediate IOL<br>The number of VE is reported<br>but only as<br>women with more than 7VEs	The rate of<br>EM for over 24h vs EM up to 24h<br>was higher for cases than controls<br>(46/92 (50%) vs 81/230 (35.2%)<br>The rate of<br>inmmediate IOL vs EM up to 24h<br>was higher for cases than controls<br>(149/198 (75%) vs 46/86 (53%)<br>No statistically significant<br>differences for<br>EM > 24h vs Immediate IOL<br>(46/86 (53%) vs 81/130 (62.3%)	8/12<br>(66.6%)
Paraiso, 2013<br>Spain	Observational study<br>Retrospective	N=115<br>n_1=Doesn't say<br>n_2=Doesn't say	G_1:Immediate IOL with oxytocin<br>G_2:Expectant up to 24h.	Outcomes: CS,<br>maternal & neonatal infection<br>Number of VEs NOT reported	No significant differences in:<br>CS or instrumental births<br>67% of women in EM<br>went into spontaneous labour<br>Only 1 case of neonatal sepsis in G_1.<br>No cases of endometritis in the study<br>Significant higher incidence in<br>intrapartum pyrexia in G_1 (IOL group)<br>They think it's because IOL process<br>is associated with longer labours and<br>more VEs than spontaneous labours<br>Length of labour not reported<br>Number of VEs not reported<br>Chorioamnionitis not defined<br>fever definition not given<br>neonatal infection not defined<br>Exact figures and proportions<br>not reported<br>Shorter length of SROM in G_1	1/12<br>(8.3%)

Supplementary table 7: Observational studies (n=5)

First author, year<br>Country	Type of study	Total N of participants<br>n per group	Intervention & comparison	Outcomes	Results	Quality
Sadeh-Mestechkin, 2016<br>Israel	Observational<br>Retrospective	N=325<br>n_1=112<br>n_2=213	G_1: Immediate IOL<br>G_2: Expectant up to 48h.	Primary: maternal or fetal signs of infection<br>chorioamnionitis<br>neonatal sepsis<br>endometritis<br>prolonged materal hospitalizacion<br>Secondary: CS<br>Reported number of VEs	No cases of neonatal infection<br>no significant differences in chorioamnionitis<br>neonatal infection:<br>G1=0 G2=0<br>Prolonged hospitalizacion:<br>G_1:2/112(1.8%) G_2:15/213(7%)	9/12<br>(75%)
Shalev, 1995<br>Israel	Observational study<br>Prospective<br>Non-randomised	N=566<br>n_1=298<br>n_2=268	G_1: IOL at 12h. with IV oxytocin<br>G_2:IOL at 72h.<br>followed by oxytocin	Unclear outcomes<br>length of SROM<br>chorioamnionitis<br>pregnancy outcome<br>chorioamnionitis, CS<br>IOL rate<br>length of hospitalization<br>Number of VEs NOT reported	No significant differences in:<br>CS, chorioamnionitis<br>Apgars at 5min, neonatal sepsis<br>Higher IOL in G_1 than G_2<br>Higher length of hospital stay in EM as women were admitted to hospital whilst waiting for labour to start<br>clinical chorioamnionitis defined as:<br>abdominal pain, fever<br>uterine irritability<br>microbiology invasion of uterine cavity from cultures taken at birth and histologic placental inflamation.<br>Not stated where temperature was taken and what was considered fever.<br>Neonatal sepsis defined as:<br>positive blood culture or cerebro-spinal fluid<br>CS:<br>G_1=14/298(4.7%) G_2=18/268 (6.7%) NS<br>Chorioamnionitis:<br>G_1=35/298 (11.7%) G_2=34/268 (12.7%)NS<br>Neonatal sepsis:<br>G_1=6/298 (2%) G_2=2/268(2.2%) NS	7/12<br>(58.3%)

Supplementary table 7: Observational studies (n=5)

First author, year<br>Country	Type of study	Total N of participants<br>n per group	Intervention & comparison	Outcomes	Results	Quality
Zamzami, 2006<br>Saudi Arabia	Observational<br>case-control	N=344<br>N_S=172<br>n_{EM}=118<br>n_{AM}=54<br>N_C=172	G_S: Divided in 2 groups chosen by consultant<br>G_{S1}:Immediate IOL with oxytocin<br>G_{S2}:Expectant up to 24h<br>G_C:Women in spontanous labour with intact membranes	Primary & secondary not stated<br>Outcomes:<br>Length of labour<br>Fetal dystress<br>Intrapartum pyrexia<br>CS<br>Apgars<br>Number of VEs NOT reported	No significant differences between control & study groups in CS<br>3 cases of intrapartum pyrexia in study groups<br>2 in AM & 1 in EM<br>0 in control	4/12<br>(33.3%)
